# Incidence of Fractures after Cardiac and Lung Transplantation: A Single Center Experience

**DOI:** 10.1155/2014/573041

**Published:** 2014-04-22

**Authors:** Aileen Hariman, Charles Alex, Alain Heroux, Pauline Camacho

**Affiliations:** ^1^Department of Endocrinology, Loyola University Medical Center, Maywood, IL 60153, USA; ^2^Department of Pulmonology/Critical Care, Advocate Christ Medical Center, Oak Lawn, IL 60453, USA; ^3^Department of Cardiology, Loyola University Medical Center, Maywood, IL 60153, USA

## Abstract

Osteoporotic fractures are well-known complications of organ transplantation. Fracture rates up to 35% have been previously reported following heart and lung transplantations. Our institutional pretransplant protocols include DXA scans, vitamin D screening, and appropriate antiresorptive therapy. We aimed to assess the incidence of fragility fractures following cardiac or lung transplantation. In a retrospective study 210 electronic medical records of patients who underwent LT (110 men, 100 women) and 105 HT (88 men, 17 women) between 2005 and 2010 were analyzed. Both clinical and radiographic fractures were recorded. DXA scans were obtained immediately after transplant. 17 out of 210 LT patients (8.0%) had fractures after transplantation and 9 out of 105 HT patients (8.6%) had fractures. The median time to the first fracture was 12 months and the mean time was 18 months for both LT and HT. In the HT recipients, the median femoral neck T score was statistically lower in the fracture group versus the nonfracture group. Similar results were seen in the LT patients. *Conclusion.* Our findings demonstrate a much lower incidence of fractures in heart and lung transplant recipients in comparison with earlier reports. Comprehensive bone care and early initiation of antiresorptive therapy are possible contributors to these improved outcomes.

## 1. Introduction

Osteoporosis and the development of fractures are well-known complications in patients following organ transplantation and affect their quality of life. In addition to risk factors such as Caucasian race, female gender, estrogen and androgen deficiency, immobilization, vitamin D deficiency, and low bone mineral density prior to organ transplantation, patients who undergo transplantation are placed on immunosuppressive therapy which may contribute to the pathogenesis and rapid development of fractures.

Patients awaiting cardiac transplantation are at risk for osteoporosis as patients with end-stage congestive heart failure have been exposed to loop diuretics resulting in a negative calcium balance. In addition, cardiac cachexia, reduced exercise levels, smoking, alcohol abuse, hypogonadism related to debilitating illness, and anticoagulation may contribute to bone loss. Low levels of 25-hydroxyvitamin D and 1,25-dihydroxyvitamin D are associated with secondary hyperparathyroidism and are common in these patients [1, 2].

Patients who undergo lung transplantation are already at increased risk for osteoporosis due to malnutrition, malabsorption from cystic fibrosis, history of smoking, hypogonadism, hypovitaminosis D, and exposure to glucocorticoids [3].

At Loyola University Medical Center, pretransplant protocols routinely include DXA scans and aggressive vitamin D screening prior to and immediately after transplantation. Antiresorptive medications are widely utilized. The aim of this study was to assess the incidence of fragility fractures following cardiac or lung transplantation.

## 2. Materials and Methods

### 2.1. Study Patients

The study population included all adults who underwent single or bilateral lung transplantation or underwent heart transplantation at Loyola University Medical Center between 2005 and 2010. All fragility fractures were included regardless of cause. Patients less than 18 years of age were excluded from the analysis.

Patients who underwent lung transplantation were grouped under different classifications by diagnosis, such as chronic obstructive pulmonary disease, interstitial fibrosis, and others. Similarly, patients who underwent heart transplantation were grouped under different classifications such as ischemic cardiomyopathy, dilated cardiomyopathy, and others.

### 2.2. Study Design

This was a retrospective observational study conducted between the years 2005–2010. Information was obtained and analyzed through electronic medical records. Both clinical and radiographic fractures through review of medical records were recorded.

The occurrence of fractures was assessed through chart review in the EPIC database, by reviewing progress notes, and radiological evidence of fracture. X-rays, MRI, CT, and DXA scans were reviewed.

Baseline data was obtained from all patients, including age at surgery, sex, BMI, prior smoking history and tobacco exposure, current use of glucocorticoids, menopausal status at the time of transplant (if applicable), and prior history of fracture.

Immunosuppression therapy for heart transplantation patients included mycophenolate, prednisone, and either cyclosporine, tacrolimus, or sirolimus. Immunosuppression therapy for lung transplantation patients included tacrolimus, prednisone, and azathioprine or mycophenolate.

### 2.3. Osteoporosis Assessment and Treatment

Patients were reviewed to assess whether they have had a DXA scan immediately following transplantation. Patients were assessed on whether they are currently using calcium and vitamin D supplementation and/or antiresorptive medication use based on objective evidence of osteopenia or osteoporosis.

### 2.4. Laboratory Data

Patients were reviewed to assess baseline and posttransplantation serum calcium, phosphorus, bone-specific alkaline phosphatase, albumin, creatinine, PTH levels, and 25-hydroxyvitamin D.

### 2.5. Data Analysis

Descriptive statistics such as mean and SD (standard deviation) or median and IQR (interquartile range) were used to summarize patient characteristics for continuous variables, whereas frequencies and percentages were used for categorical variables. Due to the small sample sizes, Wilcoxon rank sum tests were generally used to assess differences between fractured and nonfractured subjects, with respect to continuous variables (i.e., age at transplant). Chi-square or Fisher's exact tests were used to assess the association between categorical variables (i.e., gender) and the occurrence of fracture. Data was stored in excel format, and the statistical analysis was carried out using statistical analysis software (SAS).

## 3. Results

Baseline demographics, clinical characteristics, reasons for transplantation, and results prior to and following lung transplantation are listed in Tables 1 and 2. Similar data in those who underwent heart transplantation are listed in Tables 3 and 4.

### 3.1. Lung Transplantation

Out of 210 lung transplant recipients, 17 patients (8.0%) had fractures after transplantation. The incidence of fracture in lung transplant patients in years 1 to 5 was 4.8%, 1.0%, 0.0%, 1.0%, and 1.9%, respectively. The median time to the first fracture was 12 months and the mean time was 17.88 ± 15.19 months. Out of the 210 lung transplant recipients, 100 patients were female, and 8% of female patients incurred fractures. Out of the 110 male lung transplant recipients, 8.2% of male recipients incurred fractures.

The median pretransplant lumbar spine (LS) T score did not differ in those who fractured versus those who did not (−1.60 [−2.40–0.10] versus −1.00 [−2.00–0.10], *P* = .29). The median pretransplant femoral neck T score did not differ in those who fractured versus those who did not (−2.40 [−2.70–−1.80] versus −1.50 [−2.10–−0.60], *P* = .053).

The median posttransplant LS T score did not differ in those who fractured versus those who did not (−1.50 [−2.40–−0.25] versus −1.00 [−2.00–0.00], *P* = .29). The median femoral neck T score was statistically lower in the fracture group versus the nonfracture group (−2.75 [−3.32–−2.0] versus −1.60 [−2.43–−1.00], *P* = .01).

The median age (in years) of lung transplant recipients who fractured was not significantly different than those who did not (58 [50–59] versus 56 [49–62], *P* = .96). The use of bisphosphonates in fracture and nonfracture groups did not differ in lung transplant patients (53.0% versus 50%, *P* = .78). Calcium and vitamin D use did not differ between fracture and nonfracture groups (94% versus 90%, *P* = .99). Glucocorticoid use did not differ between fracture and nonfracture groups (100% versus 91%, *P* = .37).

Fracture locations varied, including 4 hip (23.5%), 3 vertebral (17.6%), 3 rib (17.6%), 2 upper extremity (11.76%), 2 wrist (11.76%), 2 lower extremity (11.76%), and 1 foot (5.89%) fracture. None of the patients incurred multiple fractures that took place concurrently. None of these patients incurred subsequent fractures. Etiology of those who fractured after transplant included 8 patients with chronic obstructive pulmonary disease (COPD), 5 with idiopathic pulmonary fibrosis (IPF), 2 with sarcoidosis, 1 with scleroderma, and 1 with alpha 1 antitrypsin deficiency (A1AD). Out of the 11 patients who had fractures prior to lung transplantation, none had fractures following transplantation.

### 3.2. Heart Transplantation

Out of 105 heart transplant patients, 9 patients (8.6%) had fractures. The incidence of fracture in heart transplant patients in years 1 to 5 was 3.8%, 1.4%, 1.9%, 0.5%, and 0.5%, respectively. The median time to the first fracture was 12 months and the mean time was 18.41 ± 15.02 months. Out of the 105 heart transplant recipients, 17 patients were female, and 11.8% of female patients incurred fractures. Out of the 88 male transplant recipients, 8.0% of male recipients incurred fractures.

The median pretransplant LS T score did not differ in those who fractured versus those who did not (−0.80 [−1.40–0.30] versus 0.55 [−0.90–1.60], *P* = .28). The median pretransplant femoral neck T score did not differ in those who fractured versus those who did not (−1.30 [−2.20–−1.20] versus −0.50 [−1.30–0.20], *P* = .09).

The median posttransplant LS T score did not differ in those who fractured versus those who did not (−0.50 [−2.30–0.00] versus −0.20 [−1.10–1.10], *P* = .21). The median femoral neck T score was statistically lower in the fracture group versus the nonfracture group (−2.50 [−3.10–−2.30] versus −1.00 [−1.70–−0.40], *P* = .003).

The median age (in years) of heart transplant recipients who fractured was not significantly different than those who did not (61.00 [55.00–68.00] versus 55.50 [46.50–62.00], *P* = .051). The use of bisphosphonates in fracture and nonfracture groups did not differ in heart transplant patients (56% versus 39%, *P* = .48). The use of calcium and vitamin D in fracture and nonfracture groups did not differ in heart transplant patients (89% versus 89%, *P* = .99). The use of glucocorticoids in fracture and nonfracture groups did not differ in heart transplant patients (56% versus 70%, *P* = .46).

Fracture locations varied, including 3 patients with vertebral (33.3%), 2 with femur (22.2%), and 1 with a foot fracture (11.1%) alone. One patient had both vertebral and femur fractures (11.1%), 1 patient had both hip and shoulder fractures (11.1%), and 1 had humeral, tibial, and toe fractures (11.1%) concurrently. None of these patients incurred subsequent fractures. Etiology of those who fractured after transplant included 5 patients with idiopathic cardiomyopathy (ICM), 3 with dilated cardiomyopathy (DCM), and 1 with viral cardiomyopathy (VCM). Out of the 4 patients who had fractures prior to heart transplantation, none had fractures following transplantation.

## 4. Discussion

The results of our descriptive study demonstrate that fragility fractures following lung transplantation and heart transplantation occur to a lesser degree compared with prior studies.

### 4.1. Lung Transplantation

There is a paucity of available studies assessing fracture risk following lung transplantation, mainly published in the late 1990s and early 2000s. In the first year after lung transplantation, symptomatic fracture rate of 15–50% and a loss of bone mineral density (BMD) of 4% to 12% have been reported [4]. Bone mineral density loss is greatest in the first 6 months following transplantation because immunosuppressive medications induce high bone turnover and uncoupling of bone formation and resorption. Patients who are evaluated for lung transplantation have a high prevalence of low BMD and osteoporosis, increasing the risk of fracture after transplantation [5]. A prospective study in 1999 demonstrated a high incidence of fractures in the first year after lung transplantation, despite receiving therapy with calcium, vitamin D, or an agent to inhibit bone resorption within the first month. On average, there was a 1.3% loss in BMD at the lumbar spine and 2.8% loss in BMD at the femoral neck, but 50% of the patients sustained a significant decline in BMD at one or both of these sites [6]. Despite these low rates of bone loss, the fracture rate remained high, with 46% of women and 13% of men sustaining fractures. Another study from 1996 demonstrated prevalent fractures in 11% of 55 patients awaiting lung transplantation and 27% of 45 lung transplant recipients [5]. In 2000, 18% (5 out of 28) of patients suffered osteoporotic fractures within 6 to 12 months after transplantation [7].

Earlier studies demonstrated high incidences of fragility fractures despite supplemental treatment to preserve bone mass. One study demonstrated a decrease in BMD at the lumbar spine and femoral neck despite prophylactic treatment with calcium and vitamin D [7]. Other studies demonstrated high incidences of fragility fractures despite therapy with calcium, vitamin D, or antiresorptive medications [6, 7]. One study demonstrated a 47.3% deficiency of 25-OHD (<30 ng/mL), which was independently associated with a lower FEV1 and more severe B-grade rejections [8].

In our patient population, the majority of patients were treated with calcium and vitamin D, which was not statistically significant between groups. Approximately half of the patients were treated with bisphosphonate therapy in both groups, which did not differ between both groups. In a pilot study, aggressive antiresorptive therapy preserved or increased BMD prior to and following transplantation by administration of a bisphosphonate prior to and following transplantation, resulting in only 4% of symptomatic fractures, compared to our current study fracture rate of 8% [9]. The status of antiresorptive therapy prior to transplantation in our study is not known.

Similar to previous studies, a reduction in BMD was present in patients prior to and following transplantation [3, 5]. Patients in both the fracture and nonfracture groups were in the osteopenia range for BMD and did not differ significantly prior to transplantation. Patients who fractured had a statistically significant difference in femoral neck BMD following transplantation but not the lumbar spine. Consistent with multiple studies, despite antiresorptive therapy and calcium/vitamin D, bone loss persisted.

### 4.2. Cardiac Transplantation

Fragility fractures are prevalent in the heart transplant population. Studies have demonstrated an incidence of vertebral fractures ranging from 1 to 44% and osteonecrosis of the femoral head in 1–9% of heart transplant recipients [2]. In a cross-sectional study evaluating 40 patients two years after cardiac transplantation, severe osteoporosis was seen at the lumbar spine in 28% and at the femoral neck in 20% of the patients. In addition, 35% of the patients had one or more vertebral fractures [10].

Rapid bone loss occurred within the first year following heart transplantation [10]. In patients who received vitamin D and calcium supplementation, without an antiresorptive agent, the incidence of bone loss at the lumbar spine based on mean percent BMD change was 7.3% and at the femoral neck was 10.5% during the first year after cardiac transplantation, with 92% of the patients suffering a significant bone loss during this year [1].

In heart transplant patients, the incidence of fracture was highest in the first year following transplantation. It has been observed in other studies that, after the initial year, there is usually a marked decline in the cumulative glucocorticoid dose and a decrease in immunosuppressive therapy [11]. The use of steroid pulses for rejection is less likely after one year; maintenance steroids are at a lower dose, and there is an annual increase in trabecular bone that persists for a minimum of 3 years.

In our patient population, there was a statistically significant decline in femoral neck BMD following transplantation. This was not seen in the lumbar spine prior to or following transplantation. These findings were similar to a prior study in which the BMD in the femur was below in both cardiac transplant candidates and recipients [2]. Prior to transplant, the use of loop diuretics may stimulate a secondary increase in PTH causing a loss of bone density at the hip.

### 4.3. Transplantation Medications

Following transplantation, patients are placed on a drug regimen that commonly includes glucocorticoids and calcineurin inhibitors. Glucocorticoid use is a common cause of secondary osteoporosis in transplant patients, and calcineurin inhibitors have adverse effects on skeletal integrity. Both of these medications can lead to bone disease characterized by rapid bone loss and high rates of fractures [12]. The use of glucocorticoids is associated with an increased risk for the development of osteoporosis and fractures by increasing apoptosis of osteoblasts and impairing osteogenesis. It also causes an increase in osteoclastogenesis and bone resorption [13]. A large decrease in BMD occurs within the first 3–6 months due to the large doses of glucocorticoids used immediately after grafting [14]. In addition, the use of other antirejection medications such as cyclosporine and tacrolimus has been shown to cause increased osteoclastic bone resorption and osteoblastic bone formation [12, 15]. The combination of calcineurin inhibitors with glucocorticoids can have severe consequences on bone density. Even with the use of multiple immunosuppressive medications to help decrease the dosage of glucocorticoids, the occurrence of osteoporosis and fractures persists.

Numerous studies are present which illustrate the decline in bone mineral density and the incidence of osteoporosis following solid organ transplantation despite the use of calcium, vitamin D, and antiresorptive therapy. One study through Northwestern University assessed the occurrence of fractures in solid organ transplant recipients (kidney, liver, and pancreas). This demonstrated a high frequency in transplant recipients, as 27.4% of patients developed fractures 2–6 years following transplantation, although patients received less than 6 months of glucocorticoids as part of the immunosuppressive regimen [16].

There are several limitations to our analysis. The data was obtained through review of medical records. Outside DXAs were not captured in some individuals. The number of DXA scans available for review prior to transplantation was often less than that following transplantation. The observed number of fractures may be underestimated as the data regarding fracture incidence was obtained through review of imaging and self-reporting during office visits. Hormonal status was not assessed, including testosterone deficiency or the use of hormone replacement therapy.

Given the long waiting time between acceptance to the waiting list and transplantation, transplant candidates with osteoporosis should be treated aggressively so that bone health is optimized following transplantation. As glucocorticoids are a risk factor for fractures, it is important to consider treatment in patients such as bisphosphonates with low bone mass or osteopenia [6]. However, the optimal duration of antiresorptive therapy is not known [9]. It is also important to aggressively screen for vitamin D deficiency as a study in the lung transplant population demonstrated that vitamin D deficiency has been associated with higher rejection rates and infections [17]. Assessment of mobility and the use of physical therapy following transplantation are another consideration for the future.

## 5. Conclusion

Our findings demonstrate a much lower incidence of fractures following transplantation in heart and lung transplant recipients in comparison to earlier reports. Comprehensive bone care, including DXA scans, screening for vitamin D deficiency, and early initiation of antiresorptive therapy at the pretransplant or immediate posttransplant period, is possible contributors to these improved outcomes.

## Figures and Tables

**Table 1:** Comparison of those with fractures versus no fractures in the lung transplant population.

	Fracture *n* = 17	Nonfracture *n* = 193	Test statistic (df)	*P*
Demographics				
Mdn age (years) (IQR)	58 (50–59) *n* = 17	56 (49–62) *n* = 192	1772.5 (209)^1^	.96
Mdn BMI (IQR)	23.65 (21.51–25.35) *n* = 16	25.01 (20.97–27.35) *n* = 187	1381.00 (203)^1^	.27
Females	8/17 (47%)	92/192 (48%)	0.005 (1)^2^	.95
Menopause	1/8 (13%)	32/91 (35%)	1.70 (1)^3^	.26
Ex-smokers	13/17 (77%)	127/192 (66%)	0.75 (1)^2^	.39
Pretransplant fracture	0/16 (0%)	11/192 (6%)	0.97 (1)^3^	.99
Reason for transplantation				
Chronic obstructive pulmonary disease	8/17 (47%)	60/193 (31%)	2.04 (2)^2^	.36
Idiopathic pulmonary fibrosis	5/17 (29%)	64/193 (33%)		
Other	4/17 (24%)	69/193 (36%)		
Pretransplant values				
Mdn LS T score (IQR)	−1.60 (−2.40–0.10) *n* = 13	−1.00 (−2.00–0.10) *n* = 131	791.50 (144)^1^	.29
Mdn femoral neck T score (IQR)	−2.40 (−2.70–−1.80) *n* = 13	−1.50 (−2.10–−0.60) *n* = 134	678.00 (147)^1^	.053
Mdn serum calcium (mg/dL) (IQR)	8.30 (7.80–8.80) *n* = 17	8.20 (7.80–9.00) *n* = 191	1740.50 (208)^1^	.88
Mdn serum phosphorus (mg/dL) (IQR)	3.15 (2.3–3.9) *n* = 8	3.60 (3.10–4.20) *n* = 93	292.00 (101)^1^	.15
Mean serum albumin (gm/dL) (SD)	2.80 (0.45) *n* = 14	2.62 (0.60) *n* = 138	−1.11 (150)^4^	.27
Mdn serum bone-specific alk. phosphatase (mcg/L) (IQR)	—	10.10 (9.40–13.10) *n* = 13	—	—
Mdn serum parathyroid hormone (pg/mL) (IQR)	50.00 (41.00–53.00) *n* = 5	41.00 (30.00–65.00) *n* = 87	277.00 (92)^1^	.45
Mdn serum vitamin D 25-OH (ng/mL) (IQR)	29.00 (20.00–38.00) *n* = 2	28.50 (18.50–37.00) *n* = 56	64.00 (58)^1^	.85
Mdn serum creatinine (mg/dL) (IQR)	0.73 (0.60–0.94) *n* = 17	0.81 (0.70–1.00) *n* = 192	1511.00 (209)^1^	.25
Posttransplant medications				
Bisphosphonate	9/17 (53%)	95/192 (50%)	0.08 (1)^2^	.78
Calcium and vitamin D	16/17 (94%)	165/183 (90%)	0.28 (1)^3^	.99
Glucocorticoid	17/17 (100%)	175/193 (91%)	1.73 (1)^3^	.37
Posttransplant values				
Mdn LS T score (IQR)	−1.50 (−2.40–−0.25) *n* = 12	−1.00 (−2.00–0.00) *n* = 98	556.00 (110)^1^	.29
Mdn femoral neck T score (IQR)	−2.75 (−3.32–−2.0) *n* = 12	−1.60 (−2.43–−1.00) *n* = 98	376.00 (110)^1^	.01
Mdn serum calcium (mg/dL) (IQR)	8.90 (8.50–9.10) *n* = 17	8.80 (8.50–9.20) *n* = 188	1713.00 (205)^1^	.87
Mdn serum phosphorus (mg/dL) (IQR)	3.05 (2.40–3.90) *n* = 10	3.80 (3.30–4.50) *n* = 102	372.50 (112)^1^	.049
Mdn serum albumin (gm/dL) (IQR)	2.90 (2.60–3.30) *n* = 11	3.10 (2.70–3.40) *n* = 123	655.50 (134)^1^	.48
Mdn serum bone-specific alk. phosphatase (mcg/L) (IQR)	10.20 (8.95–17.40) *n* = 4	8.85 (6.40–12.90) *n* = 50	140.50 (54)^1^	.33
Mdn serum parathyroid hormone (pg/mL) (IQR)	47.00 (39.00–53.00) *n* = 11	59.50 (33.50–88.50) *n* = 104	479.00 (115)^1^	.13
Mdn serum vitamin D 25-OH (ng/mL) (IQR)	22.00 (21.00–33.00) *n* = 13	25.00 (17.00–35.00) *n* = 138	1,031.00 (151)^1^	.78
Mdn serum creatinine (mg/dL) (IQR)	1.00 (0.90–1.10) *n* = 17	0.94 (0.80–1.20) *n* = 190	1641.50 (207)^1^	.59

Note: Mdn: median, IQR: interquartile range, SD: standard deviation, and df: degrees of freedom for the test statistic (i.e., ^1^Wilcoxon rank sum test due to nonnormality or low sample size in the fracture cohort; ^2^Pearson's chi-square statistic; ^3^Fisher's exact test due to low expected frequencies; and ^4^independent *t*-test with observed normality and homogeneity). Percentages are within column.

**Table 2:** Comparison of reasons for transplantation in those with fractures versus no fractures in the lung transplant population.

	Fracture *n* = 17	Nonfracture *n* = 193
Reason for transplantation		
Chronic obstructive pulmonary disease	8/17 (47%)	60/193 (31%)
Idiopathic pulmonary fibrosis	5/17 (29%)	64/193 (33%)
Other	4/17 (24%)	69/193 (36%)
	2-Sarcoidosis 1-A1AD 1-Scleroderma	25-Cystic fibrosis 10-Sarcoidosis 7-A1AD 6-Scleroderma 6-Bronchiolitis obliterans 3-Bronchiectasis 2-Lymphangioleiomyomatosis 2-Polymyositis 2-Pulmonary arterial hypertension 1-Eosinophilic granuloma 1-Desquamative interstitial pneumonia 1-Hypereosinophilic syndrome 1-Langerhan cell histiocytosis 1-Usual interstitial pneumonia 1-Venoocclusive disease

**Table 3:** Comparison of those with fractures versus no fractures in the heart transplant population.

	Fracture *n* = 9	Nonfracture *n* = 96	Test statistic (df)	*P*
Demographics				
Mdn age (years) (IQR)	61.00 (55.00–68.00) *n* = 9	55.50 (46.50–62.00) *n* = 96	648.00 (105)^1^	.051
Mdn BMI (IQR)	23.55 (22.57–28.31) *n* = 9	24.62 (21.98–28.94) *n* = 96	453.00 (105)^1^	.79
Females	2/9 (22%)	15/96 (16%)	0.26 (1)^2^	.64
Menopause	1/2 (50%)	5/15 (33%)	0.22 (1)^2^	.99
Ex-smokers	5/9 (56%)	51/96 (53%)	0.02 (1)^2^	.99
Pretransplant fracture	0	4/95 (4%)	0.39 (1)^2^	.99
Reason for transplantation				
Ischemic cardiomyopathy (ICM)	5/9 (56%)	38/96 (40%)	1.35 (2)^2^	.51
Dilated cardiomyopathy (DCM)	3/9 (33%)	49/96 (51%)		
Other	1/9 (11%)	9/96 (9%)		
Pretransplant values				
Mdn LS T score (IQR)	−0.80 (−1.40–0.30) *n* = 3	0.55 (−0.90–1.60) *n* = 42	44.00 (45)^1^	.28
Mdn femoral neck T score (IQR)	−1.30 (−2.20–−1.20) *n* = 3	−0.50 (−1.30–0.20) *n* = 45	33.50 (48)^1^	.09
Mdn serum calcium (mg/dL) (IQR)	8.90 (8.20–9.10) *n* = 9	8.90 (8.40–9.20) *n* = 95	448.50 (104)^1^	.79
Mdn serum phosphorus (mg/dL) (IQR)	4.30 (3.40–4.50) *n* = 9	3.65 (2.70–4.20) *n* = 92	553.00 (101)^1^	.26
Mean serum albumin (gm/dL) (SD)	3.10 (2.40–3.20) *n* = 9	3.00 (2.50–3.50) *n* = 93	432.50 (102)^1^	.72
Mdn serum bone-specific alk. phosphatase (mcg/L) (IQR)	*n* = 0	8.75 (6.50–11.0) *n* = 2	—	—
Mdn serum parathyroid hormone (pg/mL) (IQR)	*n* = 0	104.00 (29.50–153.50) *n* = 13	—	—
Mdn serum vitamin D 25-OH (ng/mL) (IQR)	30.50 (25.00–36.00) *n* = 2	26.00 (6.00–34.50) *n* = 9	13.50 (11)^1^	.73
Mdn serum creatinine (mg/dL) (IQR)	1.20 (1.00–1.40) *n* = 9	1.40 (1.10–1.67) *n* = 95	381.50 (104)^1^	.30
Posttransplant medications				
Bisphosphonate	5/9 (56%)	37/96 (39%)	0.99 (1)^2^	.48
Calcium and vitamin D	8/9 (89%)	84/94 (89%)	0.002 (1)^2^	.99
Glucocorticoid	5/9 (56%)	67/96 (70%)	0.77 (1)^3^	.46
Posttransplant values				
Mdn LS T score (IQR)	−0.50 (−2.30–0.00) *n* = 7	−0.20 (−1.10–1.10) *n* = 58	171.50 (65)^1^	.21
Mdn femoral DXA T score (IQR)	−2.50 (−3.10–−2.30) *n* = 7	−1.00 (−1.70–−0.40) *n* = 57	93.00 (64)^1^	.003
Mdn serum calcium (mg/dL) (IQR)	8.70 (8.20–9.20) *n* = 9	8.90 (8.50–9.10) *n* = 94	397.50 (103)^1^	.41
Mdn serum phosphorus (mg/dL) (IQR)	3.60 (3.40–3.80) *n* = 9	3.60 (3.10–4.40) *n* = 94	418.50 (103)^1^	.57
Mean serum albumin (gm/dL) (SD)	3.00 (2.00–3.00) *n* = 9	3.10 (2.80–3.40) *n* = 92	289.50 (101)^1^	.04
Mdn serum bone-specific alk. phosphatase (mcg/L) (IQR)	16.60 (10.30–22.30) *n* = 4	13.70 (9.20–15.50) *n* = 13	38.00 (17)^1^	.87
Mdn serum parathyroid hormone (pg/mL) (IQR)	68 (5–78) *n* = 5	100 (48–133) *n* = 27	49.00 (32)^1^	.09
Mdn serum vitamin D 25-OH (ng/mL) (IQR)	25.50 (24.50–29.50) *n* = 8	19.00 (14.50–28.50) *n* = 36	211.50 (44)^1^	.35
Mdn serum creatinine (mg/dL) (IQR)	1.50 (1.00–1.70) *n* = 9	1.40 (1.00–1.83) *n* = 93	470.50 (102)^1^	.94

Note: Mdn: median, IQR: interquartile range, SD: standard deviation, and df: degrees of freedom for the test statistic (i.e., ^1^Wilcoxon *W* due to low sample size in the fracture cohort and ^2^Fisher's exact test due to low expected frequencies). Percentages are within column.

**Table 4:** Comparison of reasons for transplantation in those with fractures versus no fractures in the heart transplant population.

	Fracture *n* = 17	Non-Fracture *n* = 193
Reason for Transplantation		
Ischemic Cardiomyopathy (ICM)	5/9 (56%)	38/96 (40%)
Dilated Cardiomyopathy (DCM)	3/9 (33%)	49/96 (51%)
Other	1/9 (11%)	9/96 (9%)
	-1: Viral Cardiomyopathy	-2-Marfan's Syndrome -2-Familial DCM -2-Restrictive Cardiomyopathy -2-Viral Cardiomyopathy -1-Redo Right Ventricular Failure

## Abbreviations

DXA: Dual energy X-ray absorptiometry
LS: Lumbar spine
PTH: Parathyroid hormone
25-OHD: 25-Hydroxyvitamin D
COPD: Chronic obstructive pulmonary disease
IPF: Idiopathic pulmonary fibrosis
CF: Cystic fibrosis
A1AD: Alpha 1 antitrypsin deficiency
BO: Bronchiolitis obliterans
LAM: Lymphangioleiomyomatosis
PAH: Pulmonary arterial hypertension
DCM: Dilated cardiomyopathy
ICM: Ischemic cardiomyopathy
VCM: Viral cardiomyopathy
RCM: Restrictive cardiomyopathy
RV: Right ventricular
SD: Standard deviation
IQR: Interquartile range
Mdn: Median
df: Degrees of freedom.
